# Pancreatic Cancer Cell Lines Can Induce Prostaglandin E2 Production from Human Blood Mononuclear Cells

**DOI:** 10.1155/2011/741868

**Published:** 2011-07-12

**Authors:** Svitlana P. Grekova, Assia Angelova, Laurent Daeffler, Zahari Raykov

**Affiliations:** Infection and Cancer Program, Abteilung F010 and Inserm U701, Deutsches Krebsforschungszentrum, 69120 Heidelberg, Germany

## Abstract

Accumulating evidence suggests an important role for cyclooxygenase-2 (COX-2) in the pathogenesis of a wide range of malignancies. The protumorigenic properties of COX-2 are generally thought to be mediated by its product, PGE_2_, which is shown to promote tumor spread and growth by multiple mechanisms but most importantly through modulation of the local immune response in the tumor. Pancreatic tumor cells produce various amounts of PGE_2_, some of them being even deficient in COX enzymes or other PGE_2_ synthases. Here we describe that, beside pancreatic tumor cells or stromal fibroblasts, human peripheral blood mononuclear cells can also produce PGE_2_ upon coculture with pancreatic cancer cells. Stimulating of cellular cPLA2 within PBMCs by secreted factors, presumably sPLA2, from tumor cells appeared crucial, while the direct contact between PBMCs and PDACs seemed to be dispensable for this effect. Our data is emphasizing the complex interactions participating in the formation of the tolerogenic immune milieu within pancreatic tumors.

## 1. Introduction

Pancreatic ductal adenocarcinoma (PDAC) is one of the most lethal gastrointestinal malignancies. It is the fourth most frequent cause of cancer-related deaths in North America, the sixth in Europe, and the fifth in the UK [1].

Beside surgery and chemotherapy, cyclooxygenases (COX)—the constitutive (COX-1) and the inducible (COX-2)—have been investigated as targets for treatment and prevention of pancreatic cancer since their expression seems to correlate with poor prognosis in PDAC patients [2, 3]. The two forms of COX catalyze the conversion of arachidonic acid (produced by the activity of phospholipase A2 and other enzymes) into prostaglandins [4]. One of the main representatives of prostaglandins is PGE_2_, characterized as a mediator with suppressive activity at several levels of the immune response [5]. It selectively impairs the production of interleukin-2 (IL-2) and interferon-*γ* (IFN*γ*) in T cells, blocks the production of dendritic-cell- (DC-) produced proinflammatory cytokines, including IL-12p70, and induces IL-10 production [6–8]. Earlier results suggest an involvement of COX-2 in the control of tumor-dependent angiogenesis and growth in certain pancreatic cancers and provide the rationale for inhibition of the COX pathway as an effective therapeutic approach for pancreatic tumors [3, 9]. In addition, cyclooxygenase-2 (COX-2) overexpression in transgenic mice induces a sequence of metaplasia-dysplasia events leading to neoplastic transformation in the exocrine pancreas [10].

Several works have focused on the production of PGE_2_ and the activity of enzymes involved in the earlier steps of prostaglandin production in pancreatic cancer [11, 12]. Diagnostically, PGE_2_ levels have been shown to be helpful to distinguish early intraductal papillary neoplasms (high PGE_2_) from cystic neoplasms (low PGE_2_) in patients with known mucinous lesions in the pancreas [13]. Investigations report that either COX-1 or both COX-1 and COX-2 genes can be silenced in pancreatic cancers. Furthermore, recent evidence supports that pancreatic cancers lacking COX enzymes can exploit stromal fibroblasts as an exogenous source of prostaglandins and PGE_2_ in particular [11, 14]. Such fibroblasts named also pancreatic stellate cells (PSCs) promote the progression of pancreatic cancer by increasing cancer cells' proliferation and invasion, as well as by protecting them from radiation- and gemcitabine-induced apoptosis. However, it has to be noted that, beside PSCs, other cells types such as macrophages, dendritic cells, and lymphocytes that infiltrate pancreatic carcinoma tissue actively participate in the formation of the cellular and cytokine milieu within the tumor [15]. These cells are considered to be quickly suppressed by the tolerogenic microenvironment of the PDAC tumor, for which PGE_2_ seems to play an important role [16]. In addition, recent work showed that PGE_2_ present in the PDAC patients' plasma was involved in the induction of semimature DC phenotype that does not allow these cells to function as professional antigen-presenting cells [17]. 

In our work, we describe that the culture of human pancreatic cancer cells (or their conditioned media) with peripheral blood mononuclear cells induces the release of PGE_2_ irrespective of the COX status and PGE_2_ producing capacity of the PDAC cell line used in the assay.

## 2. Materials and Methods

### 2.1. Cells and Treatment

Human pancreatic carcinoma cell lines were obtained from ATCC (Manassas, VA) and grown in RPMI 1640 (BxPC-3, T3M-4, AsPC-1) or DMEM (MiaPaCa-2) supplemented with 10% fetal calf serum (FCS), penicillin (100 U/mL), and streptomycin (100 *μ*g/mL).

Buffy coats of randomly selected healthy donors were obtained from the blood bank of the Heidelberg University, and peripheral blood mononuclear cells (PBMCs) were isolated by centrifugation over Histopaque 1077 gradient (Sigma, Hamburg, Germany), at 400 g, no brake, for 45 min at room temperature. Cells were cultured in RPMI supplemented with 10% FCS and penicillin (100 U/mL)/streptomycin (100 *μ*g/mL) at 37°C, in a 5% CO_2_ incubator. For corresponding experiments cells were simultaneously stimulated using LPS and ConA (Sigma) both at final concentration of 5 *μ*g/mL. 

The supernatants for either PDAC cells or PBMCs were obtained after centrifugation at 10000 rpm for 5 minutes before application onto the cells or use for PGE_2_ ELISA experiments.

The PLA2 inhibitor N-{(2S,4R)-4-(Biphenyl-2-ylmethyl-isobutyl-amino)-1-[2-(2,4-difluorobenzoyl)-benzoyl]-pyrrolidin-2-ylmethyl}-3-[4-(2,4-dioxothiazolidin-5 ylidenemethyl)-phenyl]acrylamide (Pyrrolidin 1) was obtained from Merck Biosciences, Germany and used at a final concentration of 1 *μ*M. The sPLA2 inhibitor 4-[(1-oxo-7-phenylheptyl)amino]-(4R)-octanoic acid (CAY10590) was used at final concentration of 91 *μ*M. The COX-1 inhibitor SC-560, was used at a final concentration of 10 nM. The COX-2 inhibitor NS-398 was used at a final concentration of 2 *μ*M. CAY10590, SC-560, and NS-398 were obtained from Cayman chemical, USA.

### 2.2. PGE_2_ Detection

The PGE_2_ measurement in culture media was performed using a PGE_2_ ELISA kit obtained from Enzo Life Sciences GmbH (Lörrach, Germany) following manufacturer's instructions. Experiments were repeated in triplicate with at least three different blood donors, and statistical significance was calculated using paired Student's *t*-test.

## 3. Results and Discussion

### 3.1. Pancreatic Cancer Cells Induce the Production of PGE_2_ from Human Peripheral Blood Mononuclear Cells

In our study we used several human pancreatic cancer cell lines from primary (MiaPaCa-2, BxPC-3) and metastatic origin (AsPC-1, T3M-4) that have been previously characterized to have various expression of the enzymes involved in PGE_2_ production. BxPC-3 have been formerly reported to intrinsically produce PGE_2_, since they are endowed with several active elements of the PGE_2_ enzymatic cascade, namely, cPLA_2_, COX-1, and COX-2 [12]. On the other hand in the same study MiaPaCa-2 and AsPC-1 were shown to be deficient in the expression of these enzymes and therefore unable to produce PGE_2_. The cell line T3M-4 derives from a metastatic tumor and has not been previously characterized in terms of its PGE_2_ production status. 

**Figure 1:**
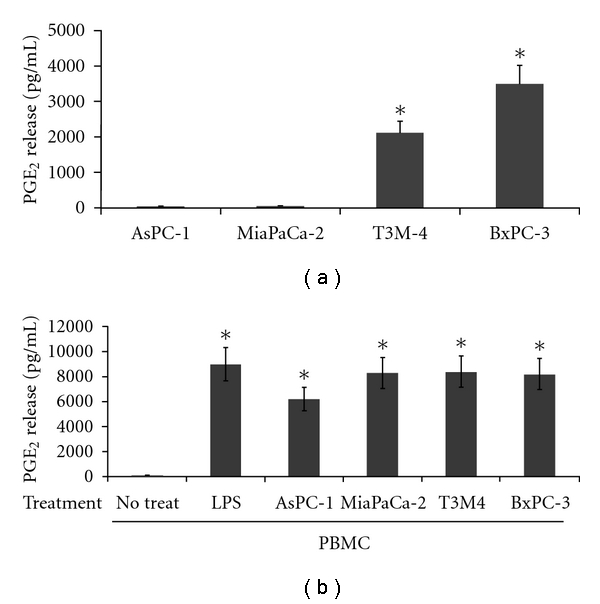
Release of PGE_2_ from pancreatic carcinoma cells and peripheral blood mononuclear cells. (a) PGE_2_ production from PDAC monocultures of cells. Cells (AsPC-1, MiaPaCa-2, T3M-4, BxPC-3) were plated in 48-well plates at a density of 2.5 × 10^4^ cells/well in 500 *μ*L of medium/well, and supernatants were collected at 48 hrs for PGE_2_ ELISA measurement. (b) PGE_2_ production from PBMCs. After purification over a Histopaque gradient mononuclear cells were plated in a 48-well plate (5 × 10^5^ cells/well) either alone or onto preseeded PDAC cultures (2.5 × 10^4^ cells/well). 48 hours after coculturing the cells, supernatants were collected and analysed for PGE_2_ expression. PBMCs treated with LPS and ConA served as controls. All experiments were repeated in triplicate with three different healthy blood donors. *Standard deviations were calculated, and results were considered significant with *P* values from Student's *t*-test below 0.05.

We tested the expression of PGE_2_ from these cells and found that AsPC-1 and MiaPaCa-2 did not produce any PGE_2_ after 48 hours of cultivation (Figure 1(a)). In contrast both BxPC-3 and T3M-4 could release significant amounts of this prostaglandin. Nonstimulated mononuclear cells from peripheral blood did not secrete PGE_2_ under normal culture conditions but could be strongly induced to release it upon treatment with LPS and ConA (Figure 1(b)). Plating of unstimulated PBMCs together with pancreatic cancer cells caused a burst of PGE_2_ activity in the medium 48 hours after coculturing the cells. The levels were comparable in all four cocultures reaching the amounts obtained after LPS/ConA stimulation (Figure 1(b)). While in the case of BxPC-3 and T3M-4, pancreatic cells could have actively contributed to the increase in PGE_2_ concentration, for AsPC-1 and MiaPaCa-2 this was rather unlikely, since they were reported as completely devoid of PGE_2_ synthases and did not produce any PGE_2_ in our experiments. Therefore, PBMCs seemed to be the only possible source of the prostaglandin within these cocultures.

### 3.2. Production of PGE_2_ from PBMCs Is Induced by Factor(s) Secreted by Pancreatic Cancer Cells

**Figure 2:**
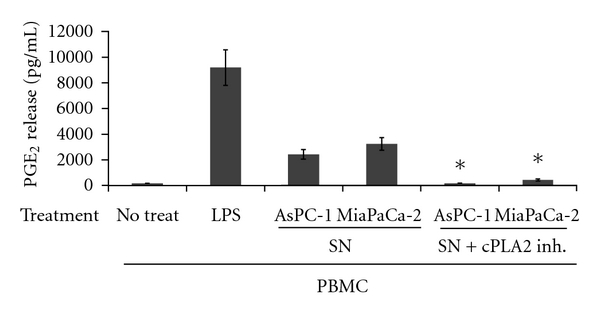
Release of PGE_2_ from peripheral blood mononuclear cells treated with PDAC supernatants. Supernatants (SN) from AsPC-1 and MiaPaCa-2 cells, derived 24 hrs after plating of the cells, were treated (SN + cPLA2 Inh.) or not (SN) with Pyrolidin 1 and applied onto cultured PBMCs (1 × 10^6^ cells/well) in a 24 well plate. Two parts of PDAC medium (400 *μ*L) were added onto one part medium overlaying the PBMCs (200 *μ*L) and 48 hrs later samples were collected and analysed for PGE_2_ expression. The experiment was repeated in duplicate with three different healthy blood donors. *Standard deviations were calculated and results were considered significant with *P* values from Student's *t*-test below 0.05.

In our next step we aimed at establishing whether PGE_2_ production may be induced by a factor secreted by tumor cells or whether it requires direct cell to cell contact. Supernatants from cultured pancreatic cancer cells were applied onto preseeded PBMCs. We selected to use conditioned media from AsPC-1 and MiaPaCa-2 cells since, as already determined above, these cells were unable to produce PGE_2_ on their own. As shown in Figure 2, LPS/ConA treatment induced a very potent PGE_2_ response from PBMCs. The supernatants of AsPC-1 and MiaPaCa-2 could also provoke the release of significant amount of the prostaglandin from PBMCs, although to a lesser extent (Figure 2). We reconfirmed that the media of AsPC-1 and MiaPaCa-2 were devoid of any PGE_2_ before being applied onto the PBMCs (data not shown). In conclusion, these data suggest that direct cell contact between PBMCs and PDACs is dispensable for PGE_2_ induction from immune cells.

The first enzymatic activity required for prostaglandin production is phospholipase A2, which among other enzymes catalyzes the release of arachidonic acid from cell membrane lipids [4]. This enzyme has several forms, the most potent being the cytoplasmic phospholipase A2 (cPLA2) [18] that can potentially be activated in PBMCs by PDACs or their secreted factors to induce PGE_2_ release. For that purpose we treated the immune cell cultures with a selective cPLA2 inhibitor (pyrolidin 1) [19] immediately after application of the conditioned AsPC-1 and MiaPaCa-2 media. The presence of the inhibitor could drastically reduce PGE_2_ production induced by pancreatic cancer supernatants (Figure 2), suggesting indeed that the inherent cPLA2 activity in PBMCs is triggered by factors secreted by pancreatic cancer cells. Given that sPLA2 may trigger PGE_2_ production, although to a lesser extent than cPLA2, and taking into account their known ability to stimulate the cPLA2 enzyme [20, 21], we evaluated the effect of different inhibitors of the PGE_2_ production cascade in cultures of human PBMCs treated with AsPC-1 and MiaPaCa-2 media. As shown in Table 1 the COX-1 enzyme was not a major player contributing to PGE_2_ production form PBMCs, while COX-2 seemed to be strongly involved in the latter process. Interestingly, both the cPLA2 and sPLA2 inhibitors seemed to have a strong inhibitory effect on PGE_2_ production, suggesting that sPLA2 enzymes most likely released from AsPC-1 and MiaPaCa-2 could represent a potential candidate to indirectly trigger PGE_2_ expression. 

Very recent study by Omura et al. [11] as well as earlier reports [14] has proposed that cancer-associated fibroblasts, representing the highest percentage of pancreatic cancer stromal cells, may serve as exogenous sources of prostaglandins for cyclooxygenase-deficient pancreatic cancers. In this study we could confirm our assumption that the immune component, which is also widely represented in PDAC stromal reaction, may represent an important alternative source of prostaglandin production in the presence of tumor cells. For instance, macrophages and dendritic cells could become such a source, since they are well known to produce high levels of PGE_2_ upon stimulation [22, 23]. 

Omura et al. [11] have also demonstrated that, in some of the clinical cancer samples and cell lines testing negative for COX-1 mRNA, certain level of expression could still be induced by epigenetic treatment with methyltransferase inhibitor 5-aza-dC or histone deacetylase inhibitor TSA. However, it is rather unlikely that in our coculture experiments PBMCs could induce such a high level of PGE-2 expression from PDACs, following mechanisms similar to chemical stimulation. 

The levels of PGE_2_ induced by supernatants of AsPC-1 and MiaPaCa-2 cells applied on the PBMCs were lower than the ones achieved in the corresponding cocultures, but one has to take into account that the concentrations of the probable secreted stimulators should be much higher in the immediate proximity of the cells than in the overlaying medium that we used for the stimulation.

## 4. Conclusion

In our work we describe a previously unknown, feature of pancreatic cancer stromal interaction, pointing that pancreatic cancer cells can induce PGE_2_ production from immune cells and further modulate host cell functions in their favor through a mechanism that may also be characteristic of other tumor entities.

## Figures and Tables

**Table 1:** PGE_2_ production from PBMCs in presence of supernatants from PDAC cell lines and specific inhibitors.

Treatment	Inhibitors				
	No inh.	cPLA2	sPLA2	COX-1	COX-2
PBMC + AsPC-1 SN^1^	100% ± 0	8.84% ± 5.64*	3.50% ± 2.94*	100% ± 81.16	1.99% ± 1.82*
PBMC + MiaPaCa-2 SN^1^	100% ± 0	14.40% ± 2.5*	6.02% ± 2.06*	68.69% ± 29.58	2.89% ± 1.58*

PGE_2_ production was measured in the coculture supernatants 48 hours posttreatment. Data are represented as percentages of PGE_2_ production, compared to that induced by PDAC supernatants on PBMCs (taken as 100%) plus. The names and concentrations of respective inhibitors are indicated in Materials and Methods. The experiment was repeated with four independent donors. Standard error was calculated using SigmaPlot 10.1 program, and the statistical significance (*) of differences was assessed by a two-tailed Student's paired *t*-test. *P* values below 0.05 were considered significant.

^1^SN-supernatant.

## Abbreviations

PDAC: Pancreatic ductal adenocarcinoma
COX: Cyclooxygenase
PBMCs: Peripheral blood mononuclear cells
PSC: Pancreatic stellate cells
PLA2: Phospholipase A2.
